# A Lecture Delivered in King's College, London, on Wednesday, 14th March, 1832

**Published:** 1832-08

**Authors:** Gilbert T. Burnett

**Affiliations:** Professor of Botany in the College and to the Medico-Botanical Society, &c. &c. &c.


### BOTANY.

*A Lecture delivered in King's College, London, on Wednesday,* 14*th March,* 1832, *by* GILBERT T. BURNETT, F.L.S., M.B. R.I. R.C.S., Professor of Botany in the College and to the Medico-Botanical Society, &c. &c. &c.

(Concluded from page 21.)

TAXONOMY, or Phy-taxonomy, the science of the laws of order or arrangement, as affecting plants, comprehends more than is generally supposed; for not only the principles of the natural and artificial schemes should in this section be considered, and the practical utility of both explained, but much physiological research will be requisite, and should be here employed in the selection of the organs from which, by their comparative immutability and relative importance in various tribes of plants, the characters of classes, orders, types, genera, and species, can most satisfactorily be drawn, and on which the distinctions of these several groups can be most securely founded. The association of varieties, the determination of species, and the demarcation of genera, which should be neither too loosely comprehensive nor too fastidiously confined, will likewise demand no less acuteness to discover their true extent, and ability to define them, than energy to resist all useless subdivisions and puerile innovations; for excessive analysis should be as sedulously shunned as excessive synthesis: both are inimical to the progress of philosophy. Furthermore, the imposition of new names, the retention or rejection of old ones, which have often been carelessly and most waywardly imposed, as well as the collation of synonymes, (a most useful but very thankless task,) will require much unobtrusive botanical and historical research. But of this enough: whatever further observations there may be to offer on the formation and revision of genera and species, on generic and specific names, their construction and correction, the study of synonymes, and so forth, I shall reserve for a future lecture.

One hundred thousand may be esteemed a very moderate computation of the existing number of vegetable species: were registered varieties and fossil plants to be enumerated, the amount would probably be much more than doubled. Now that branch of our science which teaches to distinguish with facility all these multitudinous particulars, which, without being reduced to lucid order, would seem almost infinite, and gives to its possessor the privilege of calling them each by their names, can require little to be said in commendation of its utility, or to recommend it to the favor of the natural philosopher; but time, the enemy of all our deeds, and yet by whose means we do all that is ever done, forbids me on this occasion to do more than offer the most cursory illustrations of its several departments: if, however, a proof should be required, that which we anticipated in the first section of this lecture, viz. the errors that had occurred, and the injuries that had been sustained, from not discriminating the different species of our native oaks, may be confidently referred to; and as a second illustration, should another be demanded, or one more strictly professional be preferred, we need only advert, and that only for a moment, to the uncertain state of physic before *herbcraft* (as it once was fitly called,) had assumed the nature of a science: for, of all the plants used by the ancients as medicines, and many of them would, from the accounts transmitted to our times, appear to have been possessed of important powers, how few are there of which we have positive knowledge, or to which we can now refer with any thing like tolerable certainty? In fact, of the vegetable materia medica of the ancients, we know absolutely next to nothing. Nor can this be wondered at, when we take into consideration their methods of describing plants, (if methods they can indeed be called.) Thus on reference, for example, to the works of Dioscorides, who wrote especially on herbs as medicines, we shall find that in his descriptions, when any are found, (for often there is no attempt at diagnosis,) that he compares them to each other, either totally or in their several parts; i. e. when treating of any given plant, he compares it to another; and if the type be examined, that will be found to be compared to another, and that again to a third, which he presumes to be better known and more familiar, and so on through a lengthening chain of types, ectypes, antitypes, and prototypes, until the inquirer is bewildered amidst such a variety of guides. Thus Calamintha, Acinos, Ocimastrum or Ocimoides, Erinus, Solanum, Mercurialis, and Heliotropium, are all compared to Ocimum *vulgo cognitum;* while it must be confessed that the Ocimum of Dioscorides, however familiar then to him, is now utterly unknown to us. Indeed, it would be difficult to say what vegetable it could be to which so many very dissimilar plants could be compared, or what plants they possibly could be which might be compared to it; certainly not those which now possess the names which he makes use of: for how could our Calamint, Nightshade, and Heliotrope be likened to each other, or compared with the same type, our Ocymum; i. e. flowers with distinct and syngenious anthers, with regular and irregular corollæ, to a didynamious labiate Basil? Again, Dioscorides compares Centaurium minus, Tragoriganum, Serpyllum, Marum, Polycnemon, Symphytum petræum, Ageratum, and Papaver erraticum, to Origanum; then Origanum, the type of all these plants, is compared to Hyssopus, which thus is made the clue to Origanum and all the rest; and Hyssopus is described as being "*known to all;*" but it so happens that the hyssop of Dioscorides is now not at all known, instead of being, as he says, "known to all." These examples have been selected by Gray for the purpose of shewing, and none could shew more forcibly than these, the importance of systematic arrangement, and the necessity of establishing accurate botanical diagnostic signs, by which one plant may be distinguished from another, not only when seen by the teacher, or when shewn personally to the student, but by which we may be enabled to communicate our knowledge to distant lands, and record our discoveries for the advantage of distant times. Science should be not only progressive but cumulative, and it is by system alone that we can thus, as it were, annihilate for knowledge both time and space.

The third sub-section of this branch of botany relates to the records of what was known and discovered, or misunderstood, by the ancients and our later predecessors: and we include all dissertations and fragments relating to such and collateral subjects under the general terms of Botanology or Phytochronology; that is, the history or chronicles of the science.

This mnemonic section will afford much curiously interesting matter for archæological research, as the illustration just taken from Dioscorides may serve to shew; and let it alone suffice as proof; for the length to which even our short notices of the previous departments have already reached will prevent the introduction now of any more particular examples; and therefore those which I had selected from profane and sacred sources must be postponed to a future opportunity. Here, however, I would advertise my classes, that I do not purpose entering deeply into biblical criticism on the identity or names of plants, as more than any modern pen could hope to write has been already written by Scheuchzer, Celsius, Prosper Alpinus, Forskal, and others, and, moreover, I think the subject, as it admits not of demonstration, the legitimate purpose of public lectures, can be more advantageously pursued in the closet than in the theatre; and hence I shall only conduct them to the threshold of this department, and leave them to pursue such researches in the library, where alone the fit means for their successful prosecution can be found.

We now approach the last grand section of our science, viz. Economic Botany, which includes a knowledge of the purposes to which plants either have been, are, or might be, applied as food, as medicines, or in the arts.

This knowledge is in part empirical, in part philosophic; and the more we can redeem it from empiricism, the more shall we lessen the labour and privations, the more shall we increase the comforts and the conveniences, of man. Agriculture has truly, though figuratively, been called one of the breasts of the state; and, indeed, when we consider that half an acre of arable land affords as much human food as eight hundred acres of hunting ground, the praise will not sound extreme. The application of botanical philosophy to georgical pursuits has already had a most beneficial effect; for we learn, on good authority, that since the theory of assolements, or the mutation of crops, which in this country is called the turnip husbandry, because that vegetable, by us, is the most frequently alternated with corn, has been understood and partially introduced, although prejudices prevent its entire adoption, (for there is still in this land much of the fearful inertia of ignorance to overcome,) that notwithstanding the extent of arable land has decreased, the crops produced from the lessened area have been increased, not merely in a relative but in a positive degree; i. e. an absolutely larger supply of food has been produced, although a considerably less extent of land has been tilled. And if he deserved a civic crown who caused two blades of grass to grow where only one had grown before, should that science be neglected which not only teaches this, but more; which not only increases, but, as it were, creates our food, especially by those who dread a surplus population? Science has already more than doubled, probably it will triple and quadruple, our supplies of food; for the resources of philosophy are not yet exhausted.

This increase, however, is, as I have said, far from being all that we have here to notice; for scarcely is there a plant we eat that has not been in a manner *made* (at least as an article of food) by man. The means of sustenance have been provided, but those means must be improved: plants have naturally an appetency for amelioration, otherwise they could never thus be changed; and when they are again neglected, they return to their former state, and their degeneration equally proclaims and punishes man's want of care: so sure has been the word, so strict the fulfilment of the denunciation, that, if he will not work, he shall not eat.

Wheat, the illustration first alluded to, is of this an excellent example; for so changed has it been by culture, that its native stock can nowhere now be traced, it can be recognized nowhere wild; or if, as some suppose, it has been found in Thibet, the offspring is so much altered, that many doubt the legitimacy of the descent.

Horticulture is but an offset of agriculture, and its operations are many of them copies of its original on a smaller scale; and yet, from its devotion to tender and exotic plants, it has practically become a separate pursuit; and the changes which have been wrought by art on the subjects of the gardener's care are so strange, that, even when the evidence amounts to demonstration, we can scarcely esteem them true; e. g. the cauliflower, weighing several pounds, and the cabbage, whicn often reaches half a hundredweight, are both produced from a wild plant, the leaves of which do not amount to perhaps more than half an ounce, and the peduncles of which probably not to more than half a drachm. The delicious celery, also, is formed from the acrid and poisonous smallage; the asparagus and sea-kale, so bland and mild, uncultured are austere and nauseous; wild carrots and parsnips are like sticks; and potatoes are bitter, small, and often deleterious. The changes, indeed, effected by culture in plants, are quite as great and as surprising as the changes wrought by education in mankind; nor is there a greater difference between the Briton and the Boshiesman, than between cultured and uncultured plants: for example, who would think our most luxuriant apples the offspring of the austere and verjuice crab? The cat's-head and some Normandy pippins will singly weigh from eight or ten ounces to a pound, which is often as much as the entire crop of the ancestral crab. Again, who in the wretched sloe would recognize the parent of our most luscious plums? or who, in the almond's rough and leathery coat, would discern the rudiments of the juicy peach? Yet such are the changes which culture has effected; for education, if we so may speak, is not less potent in softening constitutional austerities in plants than we know it to be in smoothing the natural asperities of men, so that to them we may apply what has so beautifully been said of us:

"Emollit mores nec sinit esse feras."

Dietetic, medical, and mercantile, are but artificial applications of this science to the purposes their names declare: of their importance, however, no proof is wanting; vegetables afford their chief food both to men and beasts, some nations wholly live on plants; and to almost all, vegetables afford their staple diet. As articles of commerce, I need only allude to one or two examples; take for instance opium and tea: the latter of which alone yields a yearly revenue of about £3,300,000; and the importations of opium amount in value to upwards of £3,000,000 per annum; tobacco likewise yields a yearly duty of £2,800,000 and the trade in opium between British India and China, although contraband, brings to the Indian government a revenue of £1,800,000 sterling. How different likewise, in a political point of view, would be the influence of this country, both in the east and in the west, if a tropical grass should no longer yield sugar; or did the Gossipium even for a year cease to clothe its seeds in cotton! it would be anything but a golden age if the poet's dream were realized, if all things could by every land be borne.

As most of the public professors of Botany have been medical men, and as it owes much of its advancement to their exertions, it is no wonder that this science should often be considered more purely medical than it really is; for the medical section has long formed its most prominent, and with the worldly its only redeeming feature. Therefore, with regard to medicine, the importance of botany scarcely need be mentioned; for granting to the full the value of mineral remedies (and no one is more willing to acknowledge their importance than myself,) still what drugs can compete with opium and cinchona? to say nothing of rhubarb, aloes, and colocynth, ipecacuan, colchicum, and camphor, senna, elaterium, and many, many others far too numerous now to catalogue; amongst which are several of our native plants, which are only left unused, unthought of, and almost unknown, because they are indigenous and common; and yet which, as I elsewhere have observed, I cannot but think have of late been too much neglected; for certain is that we compass half the globe to import a drug, the prototype of which not unfrequently solicits our hands at home. I, for one, can never think that all those plants are useless that we use not; that such countless myriads of beauteous herbs which spring profusely wild over all the deep green earth, spring oft in vain, because in vain they court man's notice and regard. I never can believe that Providence has armed the weeds of foreign lands with powers necessary for us, whilst ours are impotent to heal. I never can believe our herbs inert, whilst every plant in other climes may boast itself a physician's staff.

I have already advocated the advantages of labour, and we must all confess the peculiar advantages those men enjoy who by their station are compelled to work: we know that it is by a merciful dispensation that man has been condemned in the sweat of his brow to eat his bread; for we see that, where necessity compels not to exertions, indolence debases man almost to the level of a brute; and hence we are convinced that where most is required for the body, there most fully are developed the energies of the mind: still we cannot but perceive that many of our native plants want but to grow upon the Andes or the Alps to be sought with avidity and treated with respect. It is therefore a matter of no mean importance to which, by a resolution of the council of the Medico-Botanical Society, the attention of the scientific world has been directed, by their determination to award an honorary silver medal to the author of the best essay on the nature and properties of any indigenous plants, the medicinal powers of which have been hitherto unknown or only imperfectly recorded. Already this has produced a valuable dissertation on the medicinal properties of the holly, the powdered leaves and extract and bark of which, with probably a peculiar proximate principle called ilicine, and which by their favor I now have an opportunity of exhibiting, is shewn to be a powerful febrifuge, and said to be a worthy substitute for cinchona. A communication has likewise been made respecting the medicinal properties of the common yew (Taxus baccata), which is said to possess a power equivalent to that of digitalis over the circulating system; with this advantage, that it may be exhibited without disturbing the brain, and without any fear of producing the fatal results which occasionally follow the administration of foxglove. To me, then, it appears that this will be sinking a shaft in a very rich mine of scientific discovery, of truly philosophical investigation; and I doubt not that we shall find many of the remedies now sought from the tropics and the poles growing at our doors, and soliciting to be allowed to relieve our ills. It is an amiable idea, and one that experience goes far to verify, that wherever natural circumstances favor the production of disease, nature, (i. e. nature's God,) hath beneficently conjoined the means of cure. May not the late experiments on the holly and the willow be given here as illustrations; for, although we have so long been ploughing the Atlantic, and burdening the bosom of the deep, to bring home our harvests of Peruvian ague-cure, the ever-valuable cinchona, ilicine extracted from our hollies, and salicine from our willows, as far as experiments as yet have gone, prove equally effectual in the cure of intermittent fevers with the quinine of Peruvian bark. And where do agues most commonly prevail? where do we find remittent and intermittent fevers in the greatest frequency, and of the most fatal severity? where? but in wet low lands, in marshy and fenny districts; and where do willows love to dwell? where? but in those very fens and marshes? as if planted by the hand of Providence to relieve the diseases inseparable therefrom: and hence I cannot but regard them as the living elaboratories of nature to provide medicines for the benefit of man. (*Dissert. inaug. Med. Bot.*)

Such, gentleman, are plants, and such their attributes: the former I illustrated in the introductory lecture of the prior section; the latter, as giving rise to the three great branches of vegetable physics, organic and systematic botany, I have endeavoured, though faintly, to delineate in this. No one is more sensible than myself how faint the outline is, how imperfect the details: no one feels more fully how weak the isolated illustrations must appear, severed as they necessarily have been from contingent truths, the strength of which is common and consists chiefly in their union. To me they seem like certain buds violently plucked from this branch of the tree of knowledge, and opened perforce by art. Like such buds or bulbs, which thus discover the rudiments alone of what the seasons would develop; and like such premature examinations, which can give very insufficient ideas of the beauty and the worth of the curious gems the rude hybernacle encloses; so it seems to me, in truth, that I have done little more than present you with a branch in its wintry state, devoid of leaves, and flowers, and fruit, which are to become its ornaments and chief recommendation; have rudely opened some few of those little buds with which it is so richly studded; have presented you with nothing further than a bare and naked trunk; but which, having within it the vigour and vitality of truth, will, I trust, blossom and bear fruit some thirty, some sixty, and some a hundred fold, now that it is planted in the fertile soil of your own inquiring bosoms.

Gentlemen: before bidding you this day farewell, it may be possibly expected that I should state the reasons which have induced me to commence this season two contemporaneous courses of such very different extent; because it may perhaps be thought by some that if the most extended be not too long to teach the science, the shorter must be insufficient for that purpose. Even when I offer, in apology, my intent to discuss in one the general only, and in the other both the general and the particular details of botany, I know that some will immediately retort, (for the objection has been already raised,) that general knowledge is only another name for general ignorance. To this, however, I cannot assent; such an objection seems to me more terse than true, more sonorous than sound; for general knowledge, in the sense that I understand the term, in the sense in which I use it, means a knowledge of those laws or truths which are common or general to many individuals, or groups; and especial or particular knowledge, in like manner, means a knowledge of the truths appertaining to each individual, and which are special or particular to it or them alone. Knowledge is not the less knowledge because it is but a little knowledge, or goes but a little way; much less can that be ignorance which, if little now, is only now what most great things must once have been. Thus, for example, we all have a general knowledge of many persons and of many things with whom and with which we are not particularly acquainted, just as a general has a general knowledge of the army under his command: he knows the number and the available strength of the various regiments; he knows the proportion of horse and foot, the locality of the corps, and so forth, although he may not be particularly acquainted with each individual soldier, or even with the subaltern officers, of every battalion. With one regiment, that of which he himself is colonel, he will be particularly acquainted; but of the others, it will be sufficient that he have a general knowledge, which will enable him at any time to descend to particular inquiries, that without it could not easily be done. Thus also my colleagues and myself may have a general knowledge of the mode in which all the lectures are given within these walls, the different theatres in which the courses are delivered, the extent and object of the several studies, the number of the students in each class, and so on; although we may not know the individual pupils by name, or even by sight, of any classes except our own; and yet, with such a general knowledge, how easy is it for us to arrive here in time to be present at any particular lecture, to meet any particular professor, and to become acquainted with any particular student, which, devoid of such general knowledge, or the ready means of obtaining it, could not be done without much useless fatigue, and probably many disappointments.

From this it will be seen that I am far from disparaging, very far from despising, such general knowledge: to me it seems like guarding the outposts or garrisoning the frontier towns or fortresses, whence inroads may be made at pleasure, and with safety, into neighbouring provinces, which would be otherwise impenetrable; nor have I often found those that are possessed of the least general knowledge the most proficient in special studies: on the contrary, they who have received the most liberal education in the general sciences are frequently the most fitted to follow with advantage any particular pursuit.

"Non omnia possumus omnes," methinks the idle will immediately exclaim, and I will grant them the full force of their quotation: nay more, I will grant, without fear and without prejudice to the cause, the non-expectation as well as the non-ability: I will grant that no one is expected, as indeed no one man is able, to know all things; yet every one must know something. Universal ignorance and infinite knowledge are alike impossible to man; all are in some measure philosophers, yet, for a few who become really lovers of wisdom, how many remain for ever sophists. No person *can* entertain *too* liberal, many have too contracted views of science; few lay their foundations on too wide a scale, many build on too slight a basis. Hence arises the necessity, on all possible occasions, of inculcating a due regard for sound principles in every department of philosophy; for on first principles, as on first impressions, much subsequent importance will attach; and the mind which science does not cultivate and improve, ignorance will lay waste and over-run with errors.

Confined as we are to place and time, as it would be impossible for the natural eye to survey at once the whole of the terrestrial globe, so would it be impossible for a finite mind to behold at once the entire sphere of knowledge. The view in both instances must be partial, but of greater or less extent, according as obstacles are raised or overcome, and as the means employed are more or less adapted to the purpose: yet by an horizon would the prospect be necessarily bounded in which objects might appear trifling from their distance or vast from their obscurity, from which unknown regions the slothful would retreat with terror, but towards which the active mind would be urged by its anxiety to explore.

Let me pursue the metaphor: there is a mental as well as a corporeal eye, an intellectual as well as a material view. In both cases by an horizon is the prospect bounded, in both cases may vision be extended or obscured. Placed by nature on the plain of animal existence, man may either descend the vales of ignorance or ascend the hills of science; he may either indulge his animal propensities or cultivate his intellectual powers; the lower he proceeds, the more contracted is the circle of his knowledge; the higher he mounts, the more clear and extended the horizon that bounds his view; for the obstacles which in the one case are increased and succumbed to, in the other are lessened and subdued. Still in all cases is the horizon of the intellectual and material eye obstructed by some obstacles it *cannot*, but more frequently by others that it *will not* endeavour to surmount: for in matter only is ignorance universal, and in mind alone is knowledge boundless and complete. Therefore, the lower we look in creation the less is the capacity, and the higher we look the less the incapacity; yet in the one instance such beings attain all they are capable of acquiring, and in the other, too often, that only which they are unable to avoid. Yes, man may so pervert his talents that he may be degraded to the lowest level of humanity and become little higher than the brutes; or he may so cultivate his reason, and elevate his soul, as to be but little lower than the angels, blessed with knowledge, and thence with power. Thus, although our most extended views are necessarily confined, still they may be much contracted or enlarged; and those methods well deserve the attention of man by which his prospects through this journey of life may the most be varied, and to the utmost be extended and improved: he need never fear they will become too general or complete: and as in the regions of science as in the regions of the earth, there are certain sublime and elevated spots whence may be obtained enlarged and liberal views of surrounding districts, to these should all repair and feast their eyes with repeated visions of the richness and majesty of nature, as seen through the telescope of knowledge; but to mortal eyes the glimpse must still be partial, for now we see but as through a glass, darkly, and until we have "shuffled off this mortal coil," we never can even hope, how much soever we may often wish, to behold at once all the regions of science and the glory of them.

With regard to the subjects to be discussed in these two courses, they will be in both the same; in our daily prælections every topic will be fully treated; in the weekly summaries the various points demonstrated at length will be condensed into a more popular, and made to assume a less scholastic form. To those who attend one of the courses only, each will be in itself complete; to those who attend them both, the one will become a comment on the other: to both classes an opportunity of extra instruction will be afforded; from neither will there be anything abstracted: and therefore to the plan I do not anticipate objection. To those who entered to the autumnal lectures, I need not promise, neither from them need I ask, punctuality of attendance; but for the sake of such as may be fresh-men here, it will perhaps be right to say that they will always find me punctual to the hour, and that I shall hope to find them punctual in their attendance also. I do not, I repeat, address these observations to those who attended the previous course; for, if the lectures were in any measure worthy their attention, (and from their very regular attendance I am encouraged to hope they were.) I will confess that much of whatever merit they possessed must be attributed to this regularity in their attendance; and I would entreat every one to remember that in this case more depends upon the pupil than upon the professor, and never to forget that, excepting to a good class, it is next to impossible to give a good lecture.

Gentlemen: having, as I trust, reconciled you to my plan, it only now remains for me to thank you for the kindness with which you have received, and the attention with which you have listened to a long, and I fear in some respects a tedious lecture; to offer my grateful and sincere acknowledgments to those noblemen and gentlemen, and to those senior members of our profession, who delight to honour science by assembling within these walls; and to assure them that nothing will give us greater pleasure than to see them often here, as often as their numerous and important avocations will permit. To my junior auditors, and to those who purpose becoming industrious rather than amateur students, I have an additional word to say.

Gentlemen, I would beg you to consider yourselves as setting out upon a journey of some extent, through a country of which you are ignorant, or at least with which at present you are imperfectly acquainted; and I would beseech you to look upon me as your guide, your friend; or rather as both guide and friend; a friend who, having often trod this path before, (he knows with much pleasure to himself, he hopes with some profit to his companions,) is now willing to recommence the journey, and, by his acquaintance with the road, to lessen the labour of his fellow-travellers, and to smooth their way whenever it becomes difficult or rugged; for the course of science is sometimes arduous as the mountain path, and yet like the mountain path, it is often most beautiful where steepest. Botany, like all other studies, requires for its successful pursuit some small share both of ardour and attention, but certainly much less than has been frequently supposed; not more, perhaps far less, than many collateral sciences would seem to demand. For we ask not that entire dedication of the mind which some abstract and speculative philosophers have claimed from those who offer to become their pupils; we only ask attention.

I think we hear too much by far of the rugged road to learning, too much of "the steep where fame's proud temple stands," as if to deter, even whilst inviting, the timid yet ingenuous aspirant: the road, believe me, has many beauties in its course; the steep has many steps to ease its weary height; and they who have trod the path well know that it is not very rugged; they who have scaled the steep well know that it is not very high; the one is rugged only to the slothful, the other steep to such alone as lie grovelling at the base. Let but the will be father to the deed, and then the deed is done. Tell me not of the student's midnight toil, I know it to be rather the midnight pleasure; for what time is ever so much enjoyed as that which, redeeming from perdition more truly than any other, we may call our own: what hours are ever so dear when present, so doubly dear to memory when past, as those in which we wake and work while others sleep.

Forgive me if I am wrong, perhaps I am too hasty in my conclusions, perhaps I generalize here on insufficient grounds, on too meagre an association of particulars. There may be, in studies foreign to my pursuits, difficulties that I know not of; it becomes me, therefore, not to speak decidedly of other sciences, but to restrain my positive asseverations to my own: and yet if others truly tell of the thorny paths which lead to their shrines of knowledge, why then it must be confessed that we botanists alone of all are privileged to strew our way with flowers.

I did intend, and would fain have given some cursory illustrations of each of the several chief departments of botany, both of the general sciences of Fungology, Algalogy, Muscology, and so forth, as well as of the more special studies which, by some of our most able naturalists, have been so successfully pursued; but time forbids,—and even devoting, as I have done, this hour almost entirely to one view of the science, I have been reluctantly compelled, in my brief notices of vegetable physics, systematic and economic botany, almost entirely to omit some sections, and to pass over in others so much, so very much, most important and interesting matter, that I repent me of the boldness of my design, having fallen so grievously short in its execution. But, gentlemen, faint as the sketch has been, such are plants and such is botany; such the beings to be studied, such the schemes of study, such the subjects, such the objects of this science; a science as extensive and as important as any branch of natural knowledge; a science which, in beauty and in interest, yields to none: for although the tin box, the knife, and the glass, which are almost the whole apparatus that, as botanists, we require, look but meanly when compared with the costly machines of the mechanical philosopher, or the imposing paraphernalia of the chemist, although we cannot surprise you with the exhibition of human ingenuity or astonish the uninitiated with such brilliant experiments as the laboratory affords; although we cannot waken the heavy eye by shutting up the sun in a bottle, or rouse the dull ear of sleep by the syren tones of the girl they call invisible, still we have works of surpassing excellence to display, the handy work of one who never fails. We have experiments (i. e. observations) to make in a laboratory unequalled by human art; we have living alembics with which to work, and with us the processes of evaporation, precipitation, and distillation, are carried on to an extent, and are performed with a certainty and an exactness, utterly unapproachable by man. Yes, we also have a sun that illuminates our paths in search for truth; we also have the voice of one invisible to which we listen, a still small voice, which, although inaudible by outward ears, mentally is heard telling a tale of wonders. And thus, as I have endeavoured to convince you, we botanists are not without advantages and pleasures, some of which are peculiarly our own; and we think them not the less worthy of regard because they are enjoyments within the reach of all, or because they are privileges that can be so cheaply bought.

